# Supplementary material for: Local CpG density affects the trajectory and variance of age-associated DNA methylation changes
Source: Genome Biol. 2022 Oct 17;23:216. doi: 10.1186/s13059-022-02787-8 (PMC9575273; doi:10.1186/s13059-022-02787-8)
Supplement: Supplementary file 1 — Additional file 1: Supplementary figures Fig. S1-5 and legends. [file 13059_2022_2787_MOESM1_ESM.pdf]

Higham *et al*, Additional file 1: Supplementary figures and legends

Figure S1

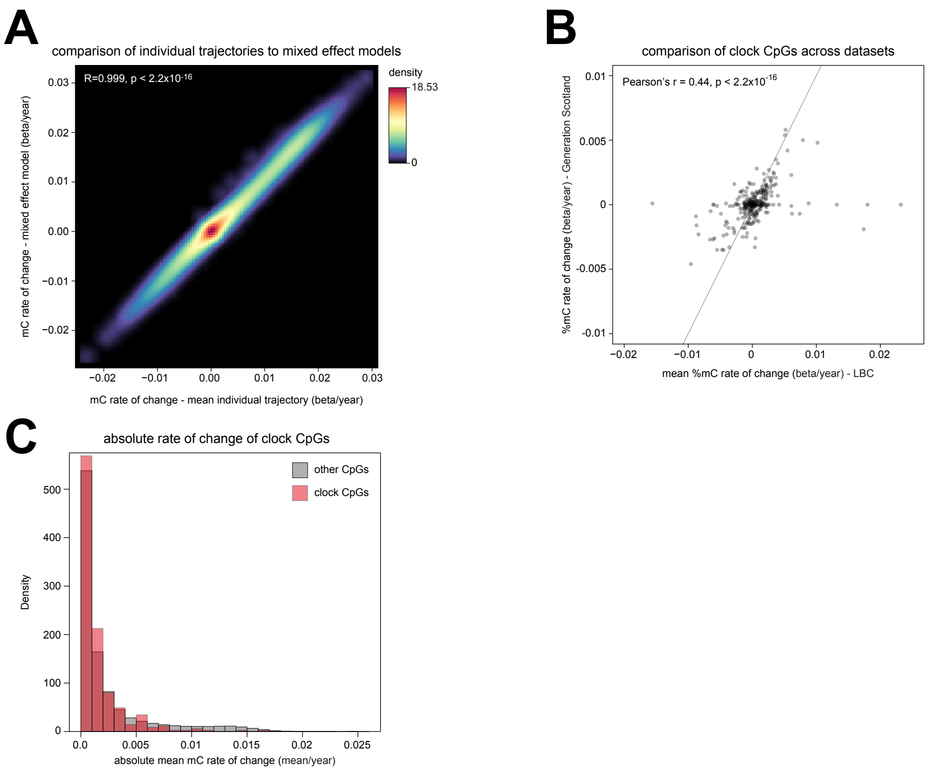
A
comparison of individual trajectories to mixed effect models
R=0.999, p < 2.2x10-16
density
18.53
0
mC rate of change - mixed effect model (beta/year)
mC rate of change - mean individual trajectory (beta/year)
B
comparison of clock CpGs across datasets
Pearson's r = 0.44, p < 2.2x10-16
%mC rate of change (beta/year) - Generation Scotland
mean %mC rate of change (beta/year) - LBC
C
absolute rate of change of clock CpGs
other CpGs
clock CpGs
Density
absolute mean mC rate of change (mean/year)

Figure S1
a) Mean individual methylation trajectories are highly correlated with the rate of change estimated from a mixed effects model. Density scatter plot of the mean methylation trajectory against the rate of change estimated from a mixed effects model with random slope. Shown are the 345,890 CpGs that could be modelled in this manner.

b) Slopes at epigenetic clock CpGs calculated from individual methylation trajectories are reproduced in a cross-sectional dataset. Scatter plot comparing the slopes calculated from the longitudinal LBC dataset to those calculated from 5,101 individuals from the cross-sectional Generation Scotland dataset. Line indicates identity.

c) Epigenetic clock CpGs show modest slopes. Histogram of the mean slope calculated from individual methylation trajectories for CpGs that are part of epigenetic clocks (red) and all other assayed CpGs (grey).

Figure S2

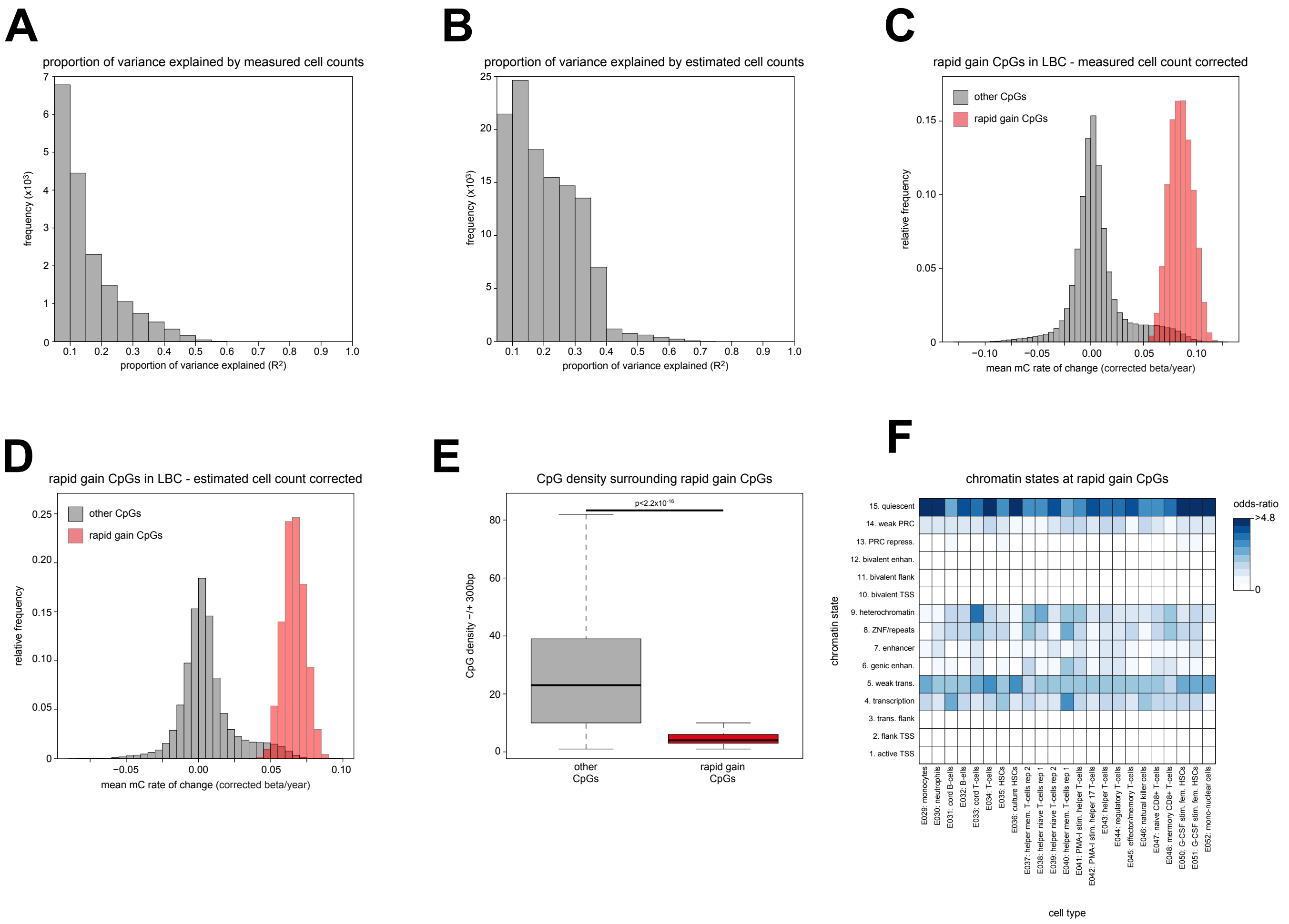
A
proportion of variance explained by measured cell counts
frequency (x10³)
proportion of variance explained (R²)
B
proportion of variance explained by estimated cell counts
frequency (x10³)
proportion of variance explained (R²)
C
rapid gain CpGs in LBC - measured cell count corrected
other CpGs
rapid gain CpGs
relative frequency
mean mC rate of change (corrected beta/year)
D
rapid gain CpGs in LBC - estimated cell count corrected
other CpGs
rapid gain CpGs
relative frequency
mean mC rate of change (corrected beta/year)
E
CpG density surrounding rapid gain CpGs
p<2.2x10⁻¹⁶
CpG density −/+ 300bp
other CpGs
rapid gain CpGs
F
chromatin states at rapid gain CpGs
odds-ratio
>4.8
0
chromatin state
15. quiescent
14. weak PRC
13. PRC repress.
12. bivalent enhan.
11. bivalent flank
10. bivalent TSS
9. heterochromatin
8. ZNF/repeats
7. enhancer
6. genic enhan.
5. weak trans.
4. transcription
3. trans. flank
2. flank TSS
1. active TSS
E029: monocytes
E030: neutrophils
E031: cord B-cells
E032: B-ells
E033: cord T-cells
E034: T-cells
E035: HSCs
E036: culture HSCs
E037: helper mem. T-cells rep 2
E038: helper niave T-cells rep 1
E039: helper niave T-cells rep 2
E040: helper mem. T-cells rep 1
E041: PMA-I stim. helper T-cells
E042: PMA-I stim. helper 17 T-cells
E043: helper T-cells
E044: regulatory T-cells
E045: effector/memory T-cells
E046: natural killer cells
E047: naive CD8+ T-cells
E048: mermory CD8+ T-cells
E050: G-CSF stim. fem. HSCs
E051: G-CSF stim. fem. HSCs
E052: mono-nuclear cells
cell type

Figure S2

a) Measured blood cell counts explain a small amount of variation in rates of change of DNA methylation with age. Histogram of proportion of variance in rates of changes in DNA methylation explained by rates of change in directly measured blood cell counts (R^2).

b) Estimated blood cell counts explain a small amount of variation in rates of change of DNA methylation with age. Histogram of proportion of variance in rates of changes in DNA methylation explained by rates of change in estimates of blood cells derived from DNA methylation data using the Houseman method (R^2).

c) Rapid gain CpGs gain methylation after correction for variation in measured blood cell counts. Histogram of the rate of change in DNA methylation (corrected beta values/year) calculated from the LBC cohort after correction for directly measured white blood cell type counts. Shown are rapid gain CpGs (red) and all other CpGs (grey).

d) Rapid gain CpGs gain methylation after correction for variation in estimated blood cell counts. Histogram of the rate of change in DNA methylation (corrected beta values/year) calculated from the LBC cohort after correction for blood cell estimates derived from DNA methylation data using the Houseman method. Shown are rapid gain CpGs (red) and all other CpGs (grey).

d) Rapid gain CpGs are found in regions of low CpG density. Boxplot comparing the CpG density -/+ 300bp from the CpG at rapid gain CpGs to all CpGs assayed. Lines=median; Box=25th–75th percentile; whiskers=1.5× interquartile range from box. P-value was calculated by Wilcoxon rank sum test.

f) Rapid gain CpGs are enriched in transcription and heterochromatin states in primary blood cells. Heatmap of the odds-ratios of rapid gain CpGs being enriched in different chromatin states in primary blood cells compared to the background of all assayed CpGs. Odds ratios derived from Fisher’s exact tests.

Figure S3

A

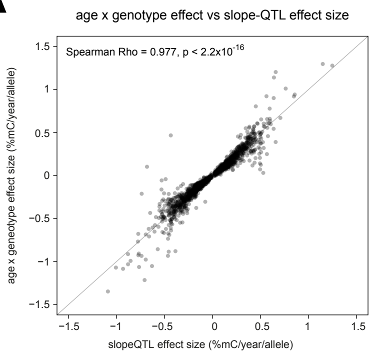

age x genotype effect vs slope-QTL effect size
Spearman Rho = 0.977, p < 2.2x10^-16
age x genotype effect size (%mC/year/allele)
slopeQTL effect size (%mC/year/allele)
1.5
1
0.5
0
−0.5
−1
−1.5

Figure S3

a) Effect sizes of slope-QTLs are significantly correlated with those calculated from age x genotype models. Scatter plots of the effect sizes calculated for slope-QTLs against the age x genotype effect calculated for the same CpG-SNP pair.

Figure S4

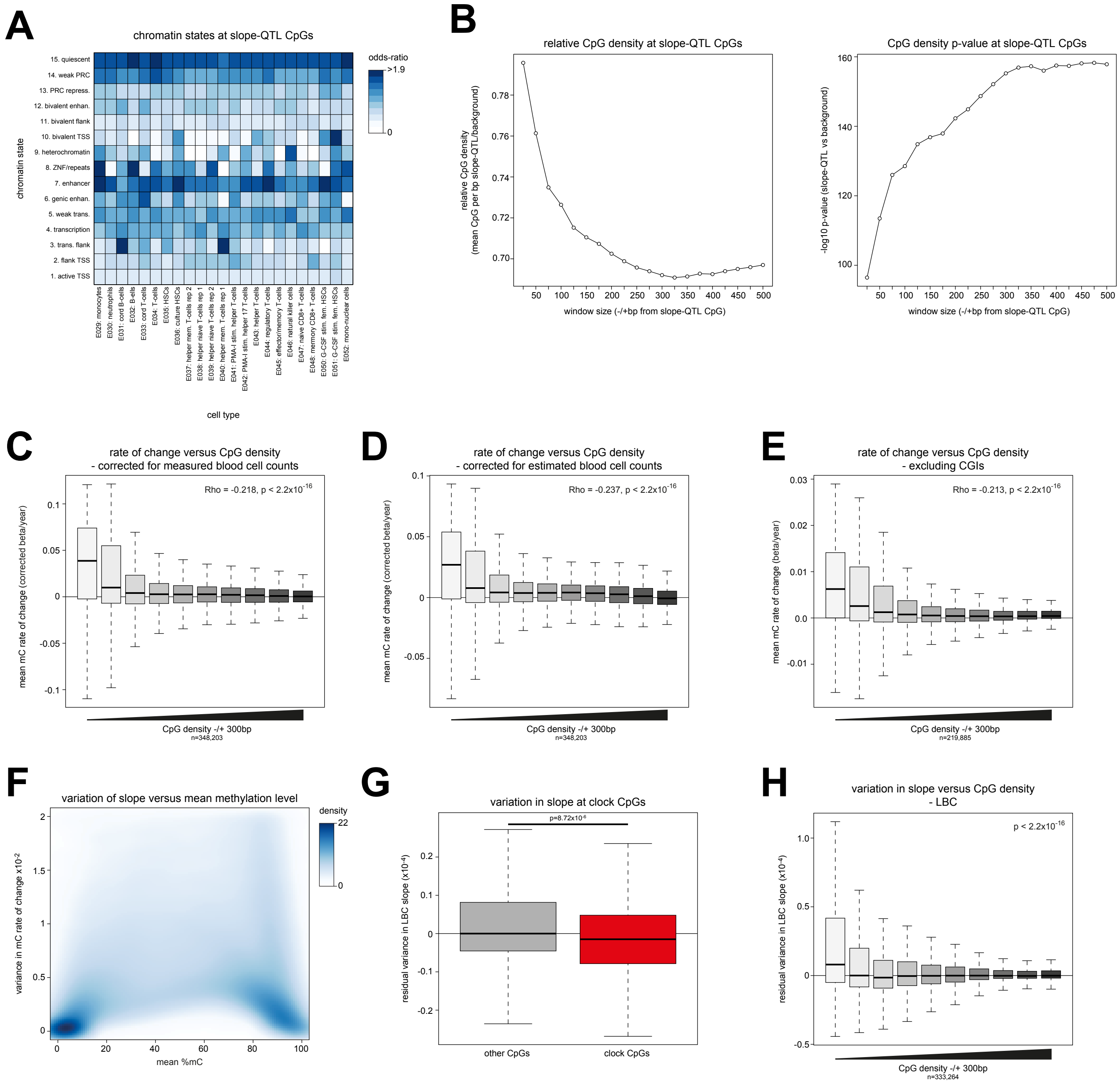

A
chromatin states at slope-QTL CpGs
odds-ratio
>1.9
0
chromatin state
15. quiescent
14. weak PRC
13. PRC repress.
12. bivalent enhan.
11. bivalent flank
10. bivalent TSS
9. heterochromatin
8. ZNF/repeats
7. enhancer
6. genic enhan.
5. weak trans.
4. transcription
3. trans. flank
2. flank TSS
1. active TSS
E029: monocytes
E030: neutrophils
E031: cord B-cells
E032: B-cells
E033: cord T-cells
E034: T-cells
E035: HSCs
E036: culture HSCs
E037: helper mem. T-cells rep 2
E038: helper niave T-cells rep 1
E039: helper niave T-cells rep 2
E040: helper mem. T-cells rep 1
E041: PMA-I stim. helper T-cells
E042: PMA-I stim. helper 17 T-cells
E043: helper T-cells
E044: regulatory T-cells
E045: effector/memory T-cells
E046: natural killer cells
E047: naive CD8+ T-cells
E048: memory CD8+ T-cells
E050: G-CSF stim. fem. HSCs
E051: G-CSF stim. fem. HSCs
E052: mono-nuclear cells
cell type
B
relative CpG density at slope-QTL CpGs
relative CpG density
(mean CpG per bp slope-QTL/background)
window size (-/+bp from slope-QTL CpG)
CpG density p-value at slope-QTL CpGs
-log10 p-value (slope-QTL vs background)
window size (-/+bp from slope-QTL CpG)
C
rate of change versus CpG density
- corrected for measured blood cell counts
Rho = -0.218, p < 2.2x10-16
mean mC rate of change (corrected beta/year)
CpG density -/+ 300bp
n=348,203
D
rate of change versus CpG density
- corrected for estimated blood cell counts
Rho = -0.237, p < 2.2x10-16
mean mC rate of change (corrected beta/year)
CpG density -/+ 300bp
n=348,203
E
rate of change versus CpG density
- excluding CGIs
Rho = -0.213, p < 2.2x10-16
mean mC rate of change (beta/year)
CpG density -/+ 300bp
n=219,885
F
variation of slope versus mean methylation level
density
22
0
variance in mC rate of change x10-2
mean %mC
G
variation in slope at clock CpGs
p=8.72x10-6
residual variance in LBC slope (x10-4)
other CpGs
clock CpGs
H
variation in slope versus CpG density
- LBC
p < 2.2x10-16
residual variance in LBC slope (x10-4)
CpG density -/+ 300bp
n=333,264

Figure S4

a) Slope-QTL CpGs are enriched in enhancer and heterochromatin states in primary blood cells. Heatmap of the odds-ratios of slope-QTL CpGs being enriched in different chromatin states in primary blood cells compared to the background of all assayed CpGs. Odds ratios derived from Fisher's exact tests.

b) CpG density differences around slope-QTLs are greatest around 300bp from the affected CpG. Line plots showing the ratio (left) and p-value(right) of differences in the CpG density at different window sizes around slope-QTL CpGs and the background of all assayed CpGs. P-values were calculated using a T-test.

c) The effect of CpG density on methylation trajectories is observed after correction for measured blood cell counts. Boxplot showing estimated rates of change in DNA methylation from the LBC cohort after correction for directly measured white blood cell counts plotted against CpG density -/+ 300bp from the CpG. The Spearman correlation, Rho, and p-value, T-test, for the association are given. For plotting, CpG density is binned into equally sized groups. Lines=median; Box=25th–75th percentile; whiskers=1.5× interquartile range from box.

d) The effect of CpG density on methylation trajectories is observed after correction for estimated blood cell counts. Boxplot showing estimated rates of change in DNA methylation from the LBC cohort after correction for Houseman estimated white blood cell counts plotted against CpG density -/+ 300bp from the CpG. The Spearman correlation, Rho, and p-value, T-test, for the association are given. For plotting, CpG density is binned into equally sized groups. Lines=median; Box=25th–75th percentile; whiskers=1.5× interquartile range from box.

e) The effect of CpG density on methylation trajectories is observed after exclusion of CpG island probes. Boxplot showing estimated rates of change in DNA methylation plotted against CpG density -/+ 300bp from the CpG when CpG island probes are excluded. The Spearman correlation, Rho, and p-value, T-test, for the association are given. For plotting, CpG density is binned into equally sized groups. Lines=median; Box=25th–75th percentile; whiskers=1.5× interquartile range from box.

f) Variance in slope between individuals is strongly related to mean methylation level. Density scatter plot of the variance in slope observed for CpGs versus their mean methylation level across all timepoints in LBC.

g) Epigenetic clock CpGs show low variance in slopes between individuals. Boxplot comparing the residualised variance in slope for CpGs that are part of the Hannum

and Horvath epigenetic clocks and all other CpGs. Lines=median; Box=25th–75th percentile; whiskers=1.5× interquartile range from box; n= 337 and 294,518 for clock and other CpGs respectively.

h) Low CpG density regions display more variable methylation slopes between individuals. Boxplot showing the variation in CpG slope across individuals from the LBC cohort plotted against CpG density -/+ 300bp from the CpG. CpG density is binned into equally sized groups. Lines=median; Box=25th–75th percentile; whiskers=1.5× interquartile range from box.

Figure S5

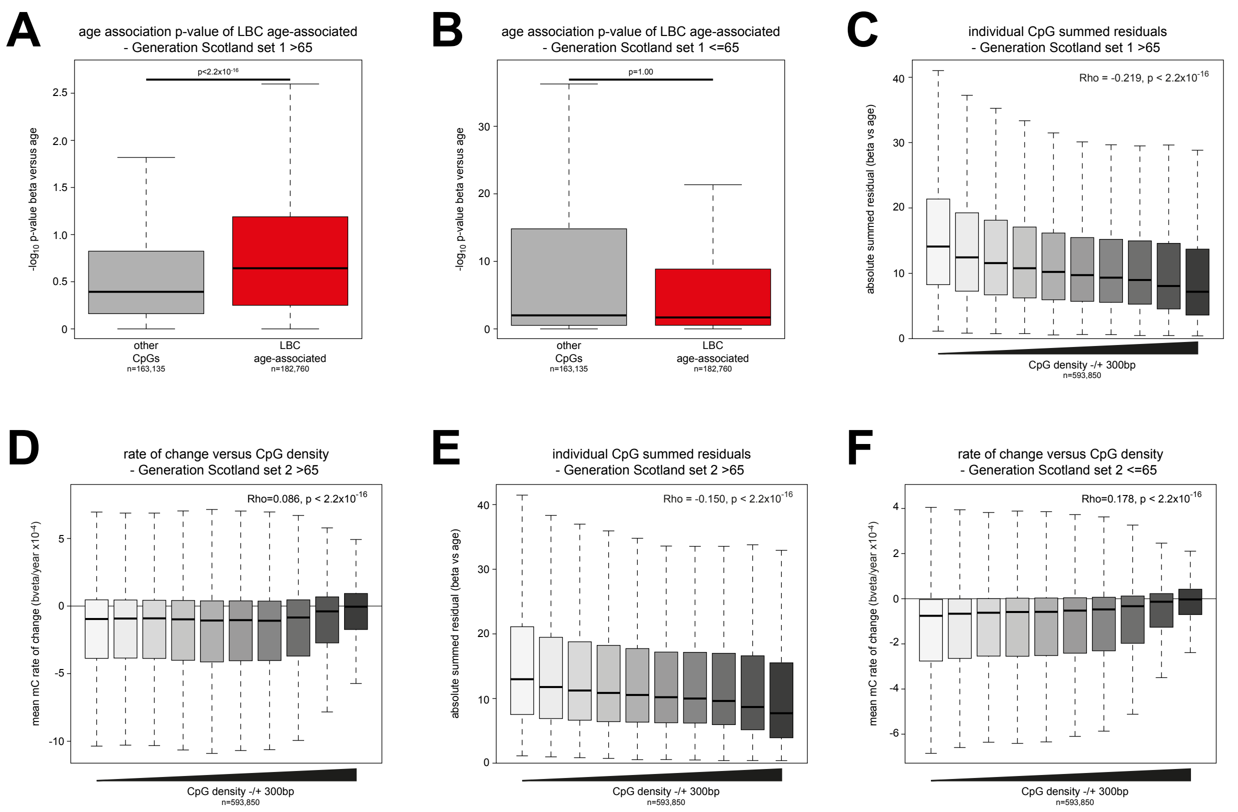
A
age association p-value of LBC age-associated
- Generation Scotland set 1 >65
p<2.2x10-16
-log10 p-value beta versus age
other CpGs
n=163,135
LBC age-associated
n=182,760
B
age association p-value of LBC age-associated
- Generation Scotland set 1 <=65
p=1.00
-log10 p-value beta versus age
other CpGs
n=163,135
LBC age-associated
n=182,760
C
individual CpG summed residuals
- Generation Scotland set 1 >65
Rho = -0.219, p < 2.2x10-16
absolute summed residual (beta vs age)
CpG density -/+ 300bp
n=593,850
D
rate of change versus CpG density
- Generation Scotland set 2 >65
Rho=0.086, p < 2.2x10-16
mean mC rate of change (bveta/year x10-4)
CpG density -/+ 300bp
n=593,850
E
individual CpG summed residuals
- Generation Scotland set 2 >65
Rho = -0.150, p < 2.2x10-16
absolute summed residual (beta vs age)
CpG density -/+ 300bp
n=593,850
F
rate of change versus CpG density
- Generation Scotland set 2 <=65
Rho=0.178, p < 2.2x10-16
mean mC rate of change (bveta/year x10-4)
CpG density -/+ 300bp
n=593,850

Figure S5

a) CpGs significantly changing with age in LBC show evidence of change in individuals >65 from Generation Scotland set 1. Boxplot of the p-values associated with linear models of CpG beta value versus age from the 406 individuals of Generation Scotland set 1 who are aged >65. Shown are $-\log_{10}$ p-values for the 182,760 CpGs significantly associated with age in LBC compared to the other 163,135 CpGs assessed in LBC. P-value from a one-sided Wilcoxon rank sum test. Lines=median; Box=25th–75th percentile; whiskers=1.5× interquartile range from box.

b) CpGs significantly changing with age in LBC do not show evidence of change in individuals <=65 from Generation Scotland set 1. Boxplot of the p-values associated with linear models of CpG beta value versus age from the 4,695 individuals of Generation Scotland set 1 who are aged <=65. Shown are $-\log_{10}$ p-values for the 182,760 CpGs associated with age in LBC compared to the other 163,135 CpGs assessed in LBC. P-value from a one-sided Wilcoxon rank sum test. Lines=median; Box=25th–75th percentile; whiskers=1.5× interquartile range from box.

c) CpG density associates with variation in age associated methylation in individuals >65 from Generation Scotland set 1. Boxplot showing absolute residual sums for models of CpG beta values fitted to age from the 406 individuals from Generation Scotland set 1 aged >65 plotted against CpG density -/+ 300bp from the CpG. The Spearman correlation, Rho, and p-value, T-test, for the association are given. For plotting, CpG density is binned into equally sized groups. Lines=median; Box=25th–75th percentile; whiskers=1.5× interquartile range from box.

d) CpG density associates with variation in age associated methylation changes in individuals aged >65 in Generation Scotland set 2. Boxplot showing estimated rates of change in DNA methylation from the 519 individuals of Generation Scotland set 2 aged >65 plotted against CpG density -/+ 300bp from the CpG. The Spearman correlation, Rho, and p-value, T-test, for the association are given. For plotting, CpG density is binned into equally sized groups. Lines=median; Box=25th–75th percentile; whiskers=1.5× interquartile range from box.

e) CpG density associates with variation in age associated methylation in individuals >65 from Generation Scotland set 2. Boxplot showing absolute residual sums for models of CpG beta values fitted to age from the 519 individuals from Generation Scotland set 2 aged >65 plotted against CpG density -/+ 300bp from the CpG. The Spearman correlation, Rho, and p-value, T-test, for the association are given. For plotting, CpG density is binned into equally sized groups. Lines=median; Box=25th–75th percentile; whiskers=1.5× interquartile range from box.

f) CpG density associates with variation in age associated methylation in individuals >65 from Generation Scotland set 2. Boxplot showing estimated rates of change in DNA methylation from the 3,931 individuals of Generation Scotland set 2 aged <65 plotted against CpG density -/+ 300bp from the CpG. The Spearman correlation, Rho, and p-value, T-test, for the association are given. For plotting, CpG density is binned into equally sized groups. Lines=median; Box=25th–75th percentile; whiskers=1.5× interquartile range from box.
